# Progress in Dermatology

**Published:** 1896-08-15

**Authors:** 


### PROGRESS IN DERMATOLOGY.

(*Continued from page* 310.)

The *Lancet*[32] insists on the importance of studying the methods of removing superfluous hairs, and discusses the various forms of depilatories. The ordinary components of these are quicklime, soda, sulphur, and arsenic, which are made into a paste; they act by desiccating and dissolving the hair shaft. Among other drugs used as depilatories, Barium sulphide and orpiment and slaked lime are mentioned, but the author thinks that all of these are unsatisfactory, and that electrolysis of the hair bulbs is the only sure treatment.

**Drug Rashes.**—Caiger[33] reports the case of a lady, aged 48, who on three occasions developed an eruption of small deeply imbedded vesicles on the palm and fingers of each hand within four days of taking a medicine containing arsenic. The patient suffered from intense feelings of irritation at the time of the appearance of the eruption; the vesicles were accompanied by œdema of the hands, and after enlarging coalesced forming bullæ. The dose of arsenic which produced the rash varied from two to five minims three times a day.

Barlow[34] describes the case of a little girl who had been treated for chorea by arsenic in three minim doses, thrice daily for some weeks, when she developed herpes zoster over one of the intercostal nerves. He refers to another case where herpes occurred after a very small dose, (one-fiftieth of a grain) given twice daily for a week only.

Cantrell[35] ascribes the development of an eruption of vesicles on both arms in a man, aged 68 years, to the taking of acetate of potash in 20 grain doses every three hours. The vesicles were accompanied by inflammation, and after a few days the condition resembled eczema. It quickly disappeared when the drug was stopped, but reappeared again in a precisely similar form when the drug was again given. This occurred on two occasions, so the author has no hesitation in saying that the eruption was undoubtedly due to the ingestion of acetate of potash.

Fordyce[36] reports the occurrence of an unusual diffuse scarletiniform eruption after the application of mercurial ointment to the pubes; free desquamation followed. He also draws attention to an erythema multiforme of the trunk and extremities, associated with conjunctivitis, which followed the ingestion of 30 grains of boric acid, which had been given daily for one month.

Haralamb[37] refers to the case of a man, aged 47 years, who suffered from general itching, succeeded by an eruption of bullæ, after the exhibition of quinine in doses varying from ½ to 1½ grammes per diem. The bullæ showed a tendency to coalesce, and then gradually dried up. At the same time an erythematous eruption appeared on the extremities. Under observation the eruption was made to appear on two occasions by administering quinine, while it disappeared immediately that drug was discontinued.

**Therapeutics.**—Cantrell[38] reviews the various skin affections which are benefited by the local application of salicylic acid. Amongst the diseases which are most benefited he mentions chronic eczema, tinea, favus, hyperidrosis, seborrhœa, warts, and nail deformities. The best form in which to employ it is either a 2 to 15 per cent. ointment or a 2 to 20 per cent. solution in water or in an emulsion of mucilage of acacia.

Bulkley[39] recommends the use of a 1 to 2 per cent. solution of permanganate of potash in water for the relief of pruritic conditions of the skin. He has found it specially useful in subacute eczema. It may cause a stinging feeling at first if the surface be abraded, but the patients speak only of the immediate relief from the itching which it affords.

Saafeld[40] gives the result of two and a half years' experience of europhen in the treatment of skin diseases. The drug is unirritating, non-poisonous, and soothing, especially to the itching and burning of eczema. Its non-poisonous qualities make it useful in the treatment of children, and it is a good substitute for iodoform after small operations. It is useful in the treatment of venereal disease.

Taylor,[41] however, reports the case of a man with a gumma of the thigh, who developed severe exudative erythema of the thigh and leg after using europhen.

Rosenthal[42] has seen good results from the external application of ichthyol in rheumatic affections, especially articular swellings; he also finds it useful in the treatment of pruritus ani and vulvæ, and in leukoplakia. He does not think that it is of any use when given internally.

For unbroken chilblains[43] the employment of a compress saturated in a decoction of bruised walnut leaves is recommended to allay the irritation. During the day either an ointment containing boracic acid 15 grains, tannin 45 grains, and vaseline 150 grains, or a powder containing starch and lycopodia a.a. 150 grains, tannin 45 grains should be used. Broken chilblains should be treated as ordinary wounds.

**Miscellaneous.**—Phillips[44] gives a statistical report of the prevalence of the various forms of skin disease in Birmingham. Out of over 21,000 cases, he finds that leprosy and favus are unknown; that tinea tonsuraus, alopecia, and scabies are less common than elsewhere, judging from the text-books; but that psoriasis is of rather more frequent occurrence.

Bulkley,[45] in a paper on sleep in its relations to diseases of the skin, insists on the importance of considering disorders of sleep in treating skin diseases. The causes of disorders of sleep may be classed as —(*a*) digestive, (*b*) toxic, (*c*) circulatory, (*d*) nervous, (*e*) psychic, and (*f*) cutaneous. The cause should be searched for, and appropriate remedies used; and when sleeplessness is due to cutaneous causes it should be treated by the proper external and internal treatment of the skin lesion, instead of resorting to hypnotics. In sleeplessness, due to itching, opium should not be given, since it is useless and even harmful; but some of the new so-called anti-neuralgic and hypnotic remedies are of service. In giving these it is often of benefit to order repeated doses at half-hour intervals until the desired effect is produced.

Boyd[46] and Manson report the case of a guinea-worm, which though broken in the foot, gave no further trouble after the injection of the surrounding tissue with a 1 in. 1,000 solution of chloride of mercury. They recommend this treatment instead of the old winding-out method, since it is equally sure, and its duration is much less. The killed worm is finally absorbed like a piece of catgut.

[32] New York Med. Journ., March 7, 1896. [33] Brit. Med. Journ., April 10, 1896, p. 967. [34] Clin. Journ., March 11, 1896, p. 311. [35] New York Med. Journ., March 21, 1896, p. 381. [36] Brit Journ. of Derm., Feb., 1896, p. 70. [37] Brit. Journ. of Derm., Feb., 1896, p. 71. [38] Ther. Gaz., April 15, 1896, p. 229. [39] Med. Rec., Feb 28 1896. [40] Brit. Med Journ. Ep, Feb. 15, 1896. [41] Brit. Journ. of Derm., Feb., 1896, p. 71. [42] New York Med. Journ., Feb. 22, 1896, p. 263. [43] Ther. Gaz, April 15, 1896, p. 250. [44] Birmingham Med. Review, March, 1896, p. 157. [45] Med. Record, Nov. 20, 1895. [46] Brit. Journ. of Derm., Feb., 1896, p. 37.

